# The interplay between TEAD4 and KLF5 promotes breast cancer partially through inhibiting the transcription of *p27*^Kip1^

**DOI:** 10.18632/oncotarget.3779

**Published:** 2015-04-22

**Authors:** Chunyan Wang, Zhi Nie, Zhongmei Zhou, Hailin Zhang, Rong Liu, Jing Wu, Junying Qin, Yun Ma, Liang Chen, Shumo Li, Wenlin Chen, Fubing Li, Peiguo Shi, Yingying Wu, Jian Shen, Ceshi Chen

**Affiliations:** ^1^ Key Laboratory of Animal Models and Human Disease Mechanisms of The Chinese Academy of Sciences and Yunnan Province, Kunming Institute of Zoology, Chinese Academy of Sciences, Kunming, Yunnan, China; ^2^ University of The Chinese Academy of Sciences, Beijing, China; ^3^ First Affiliated Hospital of Kunming Medical University, Kunming, Yunnan, China; ^4^ Department of Biochemistry, Kunming Medical University, Kunming, Yunnan, China; ^5^ Cancer Hospital, Kunming Medical University, Kunming, Yunnan, China

**Keywords:** TEAD4, KLF5, p27, hippo pathway, TNBC

## Abstract

Growing evidence suggests that YAP/TAZ are mediators of the Hippo pathway and promote breast cancer. However, the roles of YAP/TAZ transcription factor partners TEADs in breast cancer remain unclear. Here we found that TEAD4 was expressed in breast cancer cell lines, especially in triple negative breast cancers (TNBC) cell lines. TEAD4 binds to KLF5. Knockdown of either TEAD4 or KLF5 in HCC1937 and HCC1806 cells induced the expression of CDK inhibitor *p27*. Depletion of either TEAD4 or KLF5 activated the *p27* gene promoter and increased the *p27* mRNA levels. Depletion of *p27* partially prevents growth inhibition caused by TEAD4 and KLF5 knockdown. TEAD4 overexpression stimulated proliferation *in vitro* and tumor growth in mice, while stable knockdown of TEAD4 inhibited proliferation *in vitro* and tumor growth in mice. Thus TEAD4 and KLF5, in collaboration, promoted TNBC cell proliferation and tumor growth in part by inhibiting *p27* gene transcription. TEAD4 is a potential target and biomarker for the development of novel therapeutics for breast cancer.

## INTRODUCTION

Of breast cancer's four molecular subtypes, Triple negative breast cancer (ERα-, PR-, HER-2-; TNBC) is associated with comparatively poor prognosis due to a dearth of viable treatment options. This lack of therapeutics stems from many sources, one of which being the inherent complexity of TNBC etiology; TNBC is unique among the four subtypes in that it lacks the most common targeted receptors, including estrogen (ERα), progesterone (PR), and human epidermal growth factor (HER2). Thankfully, a growing number of clinical and preclinical studies have better characterized the different targets and pathways present in TNBC, including the Hippo (Hpo) pathway—a newly discovered signaling cascade that serves as a key regulator of cell proliferation and organ size [1, 2] and may play other key roles in the development and progression of cancer. Two key transcription coactivators within the Hpo pathway—YAP and TAZ—have gained increased attention. YAP and TAZ are phosphorylated by the LATS1/2-Mob1 kinase complex and are kept in cytoplasm by 14–3-3 [3–5], while unphosphorylated YAP and TAZ translocate into the nucleus and interact with transcription factors, such as TEAD1–4 [6–8], Smads [9], RunX2 [10, 11], KLF5 [12, 13], and so on.

Of the varying transcription factors interacting with YAP and TAZ, KLF5 and TEADs are particularly interesting in the study of TNBC. KLF5 is a known oncogenic transcription factor in breast cancer [12] that promotes breast cancer cell proliferation and survival partially by inducing the transcription of *FGF-BP1* and *mPGES1* [14–16]. Additionally, KLF5 inhibits the expression of CDK inhibitor *p27* in the bladder cancer cell line TSU-Pr1 [17]. Our previous studies suggest that YAP and TAZ can bind to KLF5, protect KLF5 from WWP1-mediated ubiquitination and degradation, promote the expression of KLF5 target gene *FGF-BP1*, and promote cell proliferation [12, 18]. LATS1 inhibits the expression of KLF5 and FGF-BP1 via YAP/TAZ in MCF10A and 184A1 [12, 18], collectively indicating that KLF5 can be considered as a component of the Hippo pathway. Meanwhile, TEADs act as key transcription factor partners for YAP/TAZ in terms of promoting cell proliferation, survival, migration and tumorigenesis [19], the YAP/TEAD transcription complexes regulate expression of a number of downstream target genes, for example, *CTGF* [8] and *Cyr61* [20]. The human genome encodes four highly homologous TEAD/TEF family members (TEAD1–4) that are expressed in variety of tissues [21], but recent studies suggest that TEADs may also regulate cancer development. For example, high expression levels of TEAD1 correlate with poor clinical outcomes in prostate cancer [22], while knockdown of TEAD1 decreased cell growth in PC3 and disrupted acinar formation in a 3D culture system of RWPE1 [22, 23]. Similarly, amplification and overexpression of TEAD4 were in serous fallopian tube carcinoma and testicular germ cell tumors [21, 24, 25], and TEAD4 alone promoted anchorage-independent growth in MCF10A cells [26]. However, the role of TEADs in breast cancer has not been extensively investigated, especially *in vivo*.

Alongside the transcription factors KLF5 and TEADs, further evidence also suggests that both YAP and TAZ may act as oncoproteins, promoting breast cancer tumorigenesis and metastasis. Previous studies found that YAP promotes breast cancer cell proliferation and survival [27, 28] and promotes breast cancer cell growth and progression predominately via its interactions with the TEAD transcription factors [29–31]. Similarly, other studies found TAZ to be overexpressed in TNBC [26, 32], and that TAZ expression negatively correlates with disease-free survival among breast cancer patients [33]. Knockdown of TAZ in breast cancer stem cells was found to inhibit migration and metastasis, while its overexpression in differentiated breast cancer cells was shown to induce migration and metastasis [33]. Similar to YAP, TAZ promotes breast cancer cells migration and invasion predominately through its interactions with TEAD transcription factors [8, 26, 34, 35], further suggesting a hitherto unknown connection between TEADs and breast cancer.

In this study, we investigated the expression and roles of TEADs in breast cancer and found that TEAD1 and TEAD4 are widely expressed in several breast cancer cell lines, particularly TNBC lines. We also found that TEAD4 specifically interacts with KLF5, and that knockdown of either TEAD4 or KLF5 in two TNBC cell lines upregulated the expression levels of the CDK inhibitor *p27*, while depletion of either TEAD4 or KLF5 activates the *p27* gene promoter and increased the *p27* mRNA levels. Endogenous TEAD4 and KLF5 bind to the *p27* promoter. Depletion of *p27* partially rescued TEAD4 or KLF5 knockdown induced cell growth inhibition. Finally, TEAD4 overexpression in HCC1937 significantly promotes DNA synthesis and tumor growth. Stable knockdown of TEAD4 in HCC1806 significantly inhibits DNA synthesis and tumor growth.

## RESULTS

### TEAD4 interacts with KLF5 and suppresses the *p27* gene expression in TNBC cell lines

**Figure 1:**
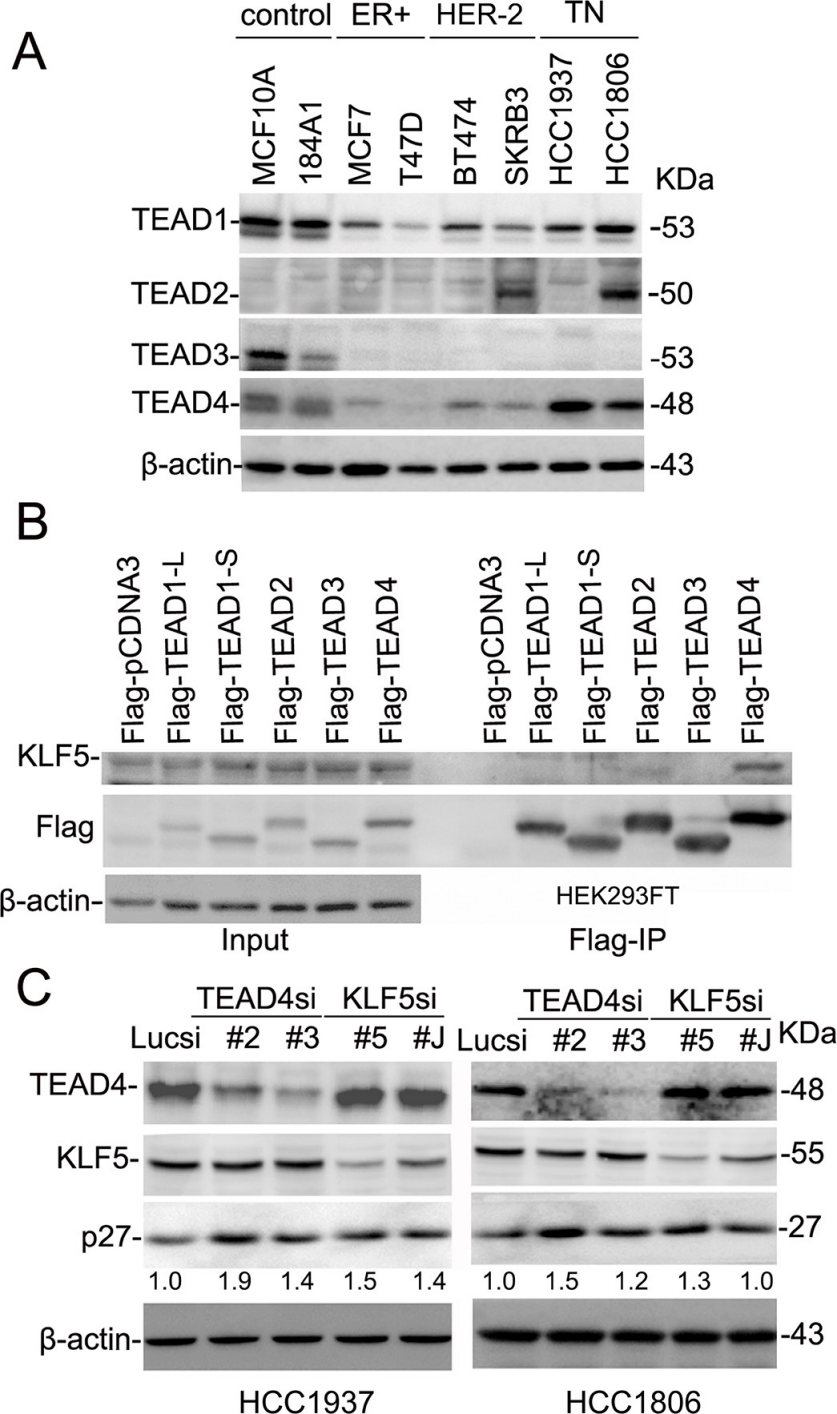
TEAD4 interacts with KLF5 and suppresses the *p27* gene expression in TNBC cell lines **A.** Protein expression levels of TEAD1–4 in breast epithelial cell lines by WB. β-actin serves as the loading control. **B.** TEAD4 specifically interacts with KLF5 in HEK293FT cells. All TEADs were tagged with Flag and immunoprecipitated with the anti-Flag antibody. Exogenous KLF5 was only immunoprecipitated by Flag-TEAD4. TEAD1-L and -S are two different TEAD1 isoforms. **C.** TEAD4 and KLF5 suppress the protein levels of *p27* in both HCC1937 and HCC1806. TEAD4 and KLF5 were silenced by two different siRNAs in both cell lines. The *p27* and β-actin protein levels were quantified by the IMAGE J software. The normalized *p27* protein levels are shown below the *p27* panel.

We first examined the protein expression levels of TEAD1–4 in two immortalized breast epithelial cell lines and six breast cancer cell lines via Western blotting (Figure 1A) to explore the role of TEADs in breast cancer. Because the protein sequences of TEAD1–4 are highly homologous to one another, we first validated TEAD1–4 antibodies (data not shown). Our examination showed that both TEAD1 and TEAD4 are widely expressed in breast cell lines, though the expression levels were higher in two basal immortalized breast epithelial cell lines and two basal TNBC cell lines as compared to ERα+ or HER-2+ breast cancer cell lines (Figure 1A). TEAD2 expression was only detected in the SKBR3 and HCC1806 lines, while TEAD3 expression was only detected in two of the immortalized breast epithelial cell lines.

Since both TEADs and KLF5 interact with YAP/TAZ, we suspected that TEADs may interact with KLF5. Co-immunoprecipitation (Co-IP) experiments showed that TEAD4 specifically interacts with exogenous KLF5 (Figure 1B), and that two TEAD1 isoforms, as well as TEAD2 and TEAD3, do not interact with KLF5. We next tested whether TEAD4 and KLF5 regulate the expression of KLF5 downstream target genes in TNBC cells. In a previous study, we demonstrated that KLF5 inhibits the expression of *p27* [17]. Here, we knocked down TEAD4 and KLF5 in HCC1937 and HCC1806 TNBC cell lines by two different siRNAs, and we observed that silencing KLF5 or TEAD4 resulted in up-regulation of *p27* protein levels in both cell lines (Figure 1C).

### TEAD4 overexpression promotes TNBC cell proliferation and tumor growth

**Figure 2:**
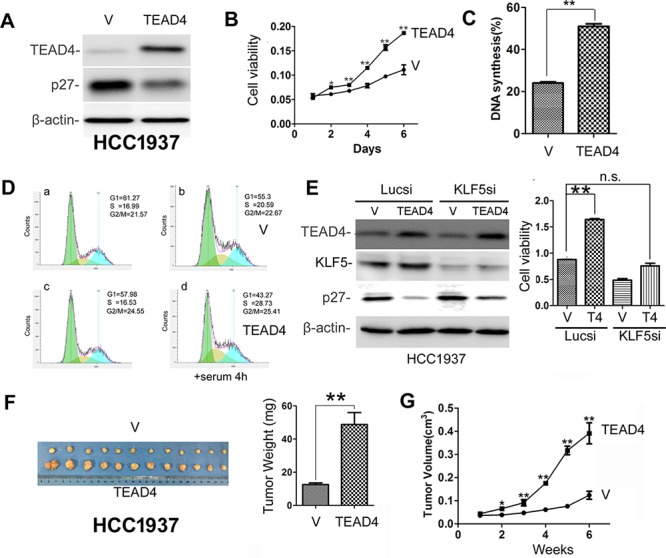
TEAD4 overexpression promotes TNBC cell proliferation and tumor growth **A.** Stable overexpression of TEAD4 decreased the *p27* protein expression level in HCC1937. **B.** TEAD4 overexpression significantly promoted HCC1937 cell growth, as determined by the SRB assay. **p < 0.01, *t*-test. **C.** TEAD4 overexpression significantly (*p* < 0.01) increased DNA synthesis in HCC1937 cells, as measured with a Click-iT EdU Alexa Fluor Imaging Kit. **D.** TEAD4 overexpression significantly increased G1/S cell cycle transition in HCC1937 cells, as measured by PI staining and flow cytometry. a&c, the cells were arrested at the G1 phase by serum starvation. b&d, the arrested cells were stimulated with 10% serum for 4 hours. **E.** TEAD4 promotes cell proliferation in part through KLF5 in HCC1937 cells. KLF5 was transiently knocked down in HCC1937-pBabe and HCC1937–pBabe-TEAD4 cells. Western blot results are shown on the left side and cell viability data are shown on the right side. **, *p* < 0.01, n.s., not significant, *t*-test. **F.** Tumor masses harvested from HCC1937-pBabe and –pBabe-TEAD4 after tumors had grown for 6 weeks. TEAD4 overexpression significantly increases xenograft weight (right panel). **G.** TEAD4 overexpression significantly promotes tumor growth in female NOD-SCID mice.

Our previous studies showed that KLF5 promotes breast cancer cell proliferation, survival and tumor growth [12, 17, 18, 41], but whether or not TEAD4 has similar functions is not entirely clear. To test the effect, we overexpressed TEAD4 in HCC1937 (Figure 2A), and as expected, stable overexpression of TEAD4 reduced the *p27* protein level (Figure 2A). We also found that TEAD4 overexpression promoted HCC1937 cell growth *in vitro* (Figure 2B). Since *p27* suppresses G1/S cell cycle transition, it is plausible that TEAD4 increases DNA synthesis in the S phase. To test this possibility, we examined DNA synthesis using the Click-iT EdU Alexa Fluor Imaging Kit in HCC1937 cells. As shown in Figure 2C and S1A, TEAD4 significantly increased the ratio of EdU-positive S phase cells in HCC1937. We further confirmed that TEAD4 promotes HCC1937 G1/S cell cycle transition by flow cytometry analysis (Figures 2D and S1B). Interestingly, when KLF5 is knocked down in HCC1937, TEAD4 overexpression did not effectively inhibit the *p27* expression and promote cell proliferation (Figure 2E). More importantly, the tumorigenesis assay showed that overexpression of TEAD4 significantly promoted the xenograft growth in NOD-SCID mice (Figure 2F–2G). The tumor weights and volumes of HCC1937-TEAD4 were significantly greater than those of HCC1937-pBabe (Figure 2F–2G).

### TEAD4 knockdown inhibited TNBC cell proliferation and tumor growth

**Figure 3:**
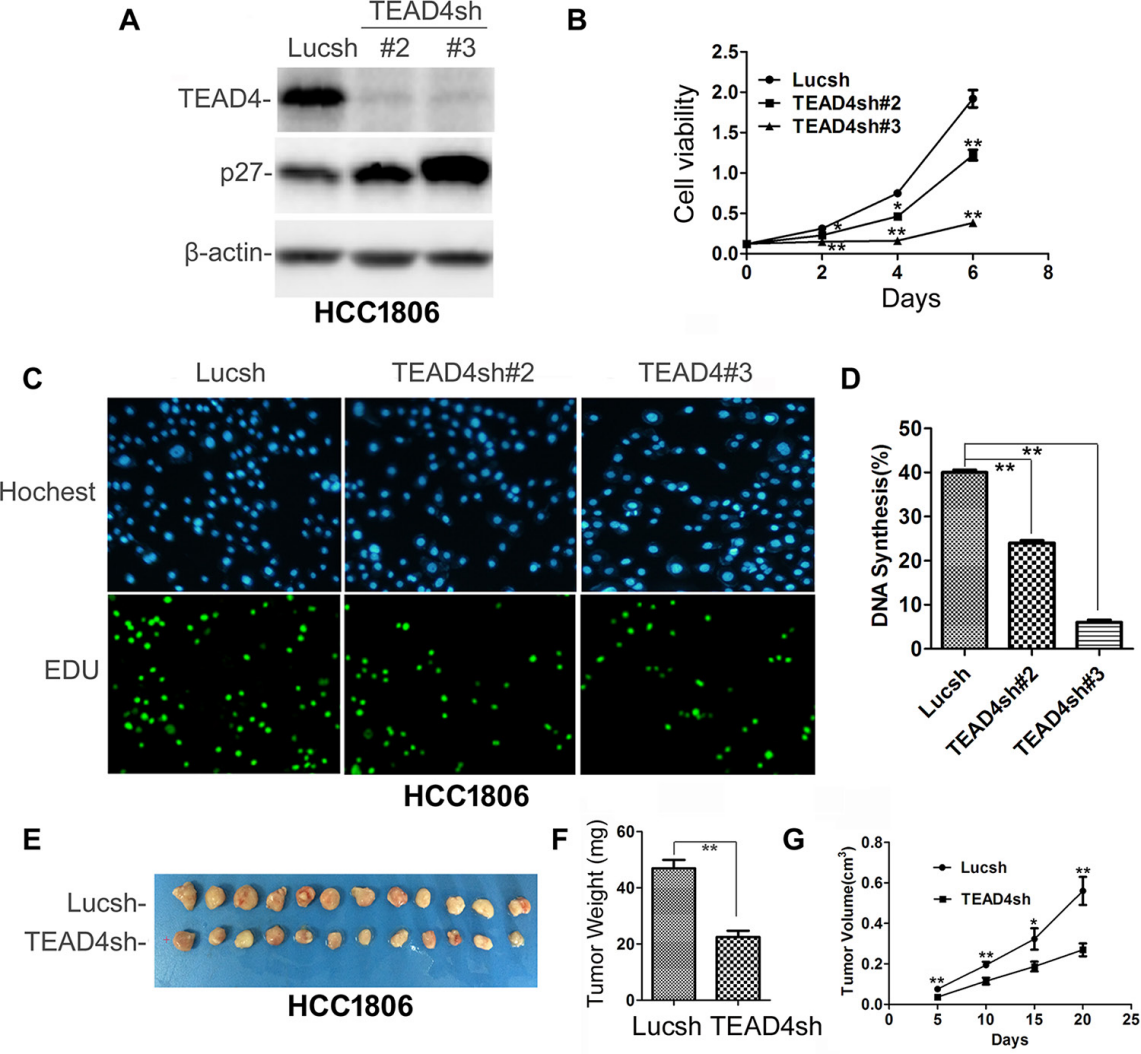
TEAD4 knockdown inhibited HCC1806 cell proliferation and tumor growth **A.** Stable knockdown of TEAD4 increased the *p27* protein level. **B.** TEAD4 knockdown significantly inhibited cell growth, as determined by the SRB assay. *p < 0.05, **p < 0.01, *t*-test. **C.** TEAD4 knockdown inhibited DNA synthesis by using the Click-iT EdU Alexa Fluor Imaging Kit. **D.** Quantitative results of panel C, **p < 0.01, *t*-test. **E.** Tumor masses harvested from HCC1806-Lucsh and –TEAD4sh#3 after tumors had grown for 21 days. **F.** TEAD4 knockdown significantly decreases xenograft tumor weight. **G.** TEAD4 knockdown significantly suppressed tumor growth in female nude mice.

To test whether endogenous TEAD4 also promotes TNBC cell proliferation and tumor growth, we stably knocked down TEAD4 in HCC1806 cells (Figure 3A) and HCC1937 (Figure S2A), and as expected, stable knockdown of TEAD4 increased the *p27* protein levels (Figures 3A and S2A) and inhibited cell growth *in vitro* (Figure 3B and S2B). Moreover, TEAD4 knockdown significantly decreased the ratio of EdU-positive cells in both HCC1806 (Figure 3C–3D) and HCC1937 (Figure S2C) cells. We also performed a tumorigenesis assay in nude mice and found that knockdown of TEAD4 significantly suppressed the xenograft growth of HCC1806 (Figure 3E–3G); indeed, both tumor weights and volumes in the HCC1806-TEAD4sh#3 group were significantly less than those of HCC1806-Lucsh group (Figure 3F–3G).

### TEAD4 and KLF5 promotes cell proliferation partially through suppressing the *p27* gene transcription in TNBC cell lines

**Figure 4:**
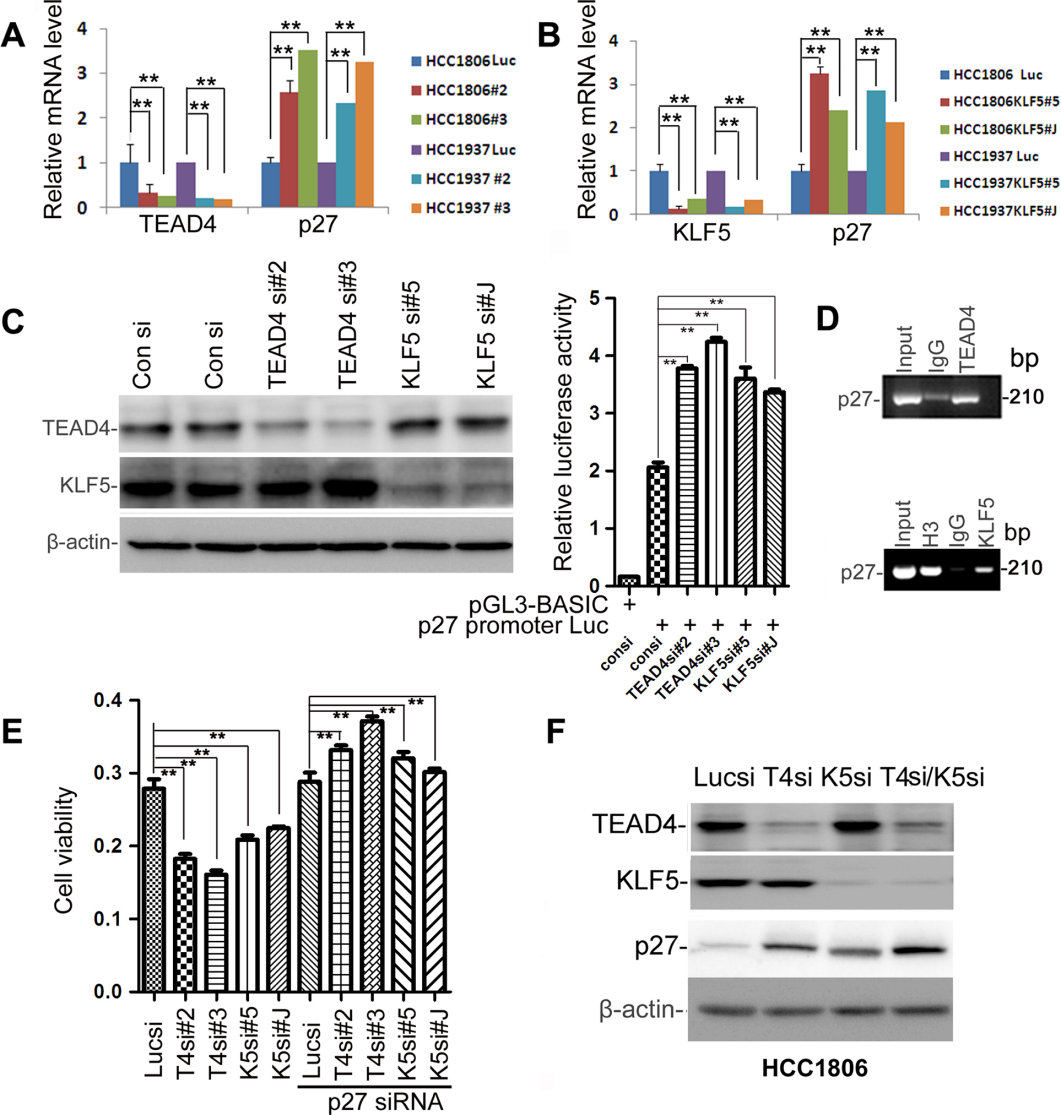
TEAD4 and KLF5 promote cell proliferation partially by suppressing the *p27* gene transcription together in TNBC cell lines **A.** Knockdown of TEAD4 by two different siRNAs upregulated *p27* mRNA levels in both HCC1937 and HCC1806, as measured by qRT-PCR. **B.** Knockdown of KLF5 by two different siRNAs upregulated *p27* mRNA levels in both HCC1937 and HCC1806, as measured by qRT-PCR. **C.** Knockdown of TEAD4 and KLF5 activated the *p27* gene promoter in HCC1806 cells. TEAD4 and KLF5 knockdown were evaluated by WB. Luciferase activities were measured by the dual-luciferase assay kit. **D.** Both TEAD4 and KLF5 bind to the *p27* gene promoter as determined by ChIP assays in HCC1806. **E.** Knockdown of *p27* rescued the TEAD4 and KLF5 knockdown-induced growth arrest in HCC1806, as determined by the SRB assay. **p < 0.01, *t*-test. **F.** Knockdown of KLF5 and TEAD4 together additively increased the *p27* protein levels in HCC1806.

Since both TEAD4 and KLF5 suppress *p27* protein expression, it is possible that the two transcription factors work together to suppress the *p27* gene transcription. To test this, we first knocked down TEAD4 and KLF5 in HCC1937 and HCC1806 cells and then examined the *p27* mRNA levels, which together showed that *p27* mRNA levels were significantly up-regulated (Figure 4A–4B). Analysis of the *p27* gene promoter identified potential TEAD4 binding sites (5′-CATTCCT-3′) and KLF5 binding sites (GC boxes), so we then performed dual-luciferase assays, which demonstrated that depletion of TEAD4 or KLF5 significantly increased the *p27* gene promoter activity (Figure 4C). We also performed ChIP assays, which showed that both TEAD4 and KLF5 bind to the endogenous *p27* gene promoter.

To determine whether TEAD4 and KLF5 promote cell proliferation through *p27*, we performed a rescue experiment in the HCC1806 cell line. We found that knockdown of either TEAD4 or KLF5 increased the *p27* protein levels and suppressed the cell growth (Figures 4E and S3). After the elevated *p27* protein levels were silenced, TEAD4 and KLF5 depletion-induced cell growth arrest were significantly rescued (Figure 4E). Finally, we demonstrated that knockdown of KLF5 and TEAD4 together additively increased the *p27* protein levels in HCC1806 (Figure 4F). These results suggest that TEAD4 and KLF5 promote cell proliferation in part by inhibiting the expression of the *p27* gene together.

### TEAD4 and KLF5 promotes cell migration

**Figure 5:**
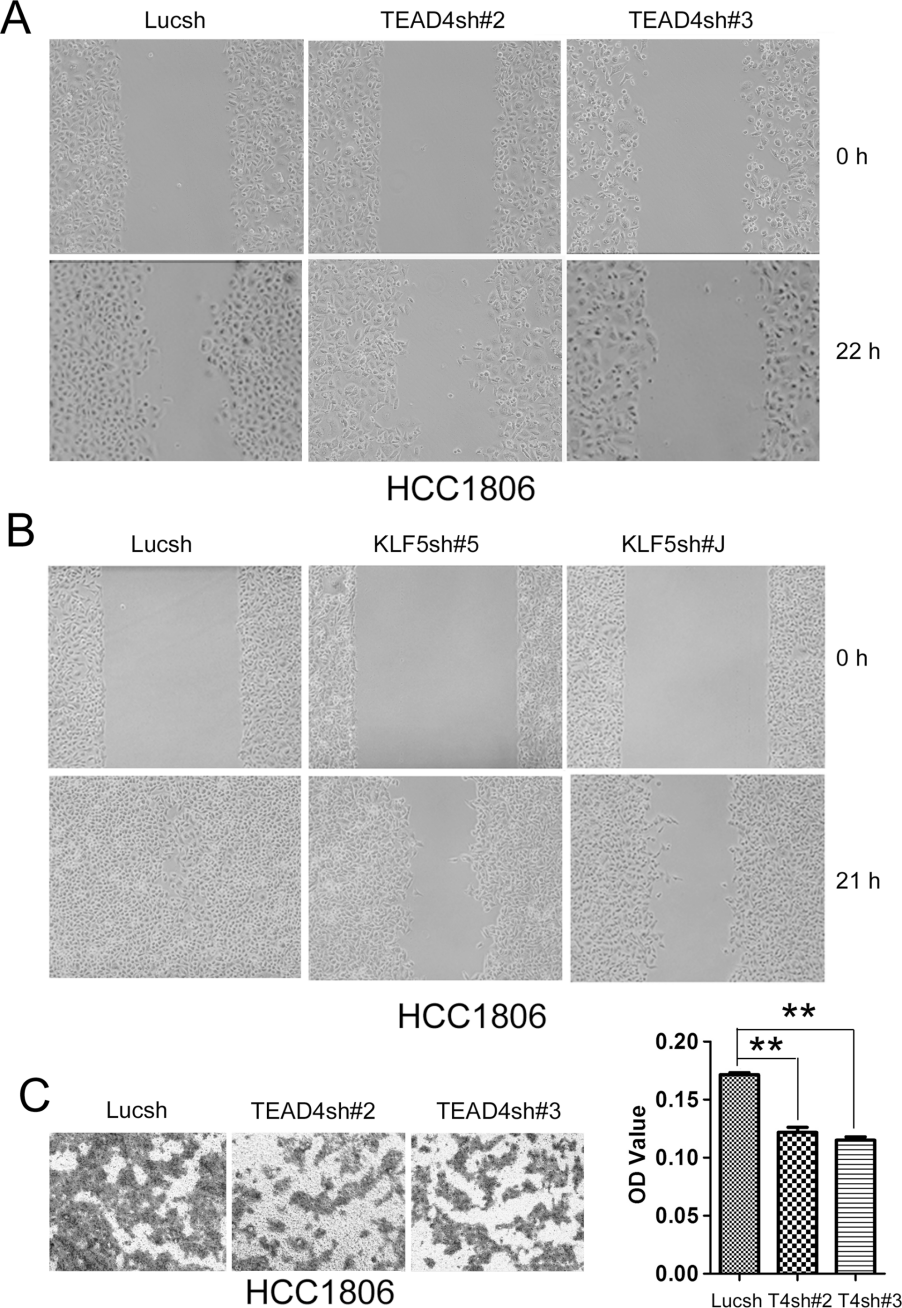
TEAD4 and KLF5 promote cell migration in HCC1806 cells **A.** Knockdown of TEAD4 by two different shRNAs decreased wound healing at 22 hours. **B.** Knockdown of KLF5 by two different shRNAs dramatically decreased wound healing at 21 hours. (note: The cell density is higher than that in panel A) **C.** Knockdown of TEAD4 by two different shRNAs decreased transwell cell migration (24 h). Quantative data is shown on the right side. **p < 0.01, *t*-test.

We further determined whether TEAD4 and KLF5 promote cell migration in TNBC cells. By wound healing assays, knockdown of either TEAD4 or KLF5 in HCC1806 decreased cell motility (Figure 5A–5B). It appears that the function of TEAD4 is weaker than KLF5 in terms of promoting cell migration. To further confirm that TEAD4 promotes cell migration, we performed transwell assays. As shown in Figure 5C, silencing TEAD4 in HCC1806 indeed significantly decreased cell migration.

### TEAD expression in human breast tumors

**Figure 6:**
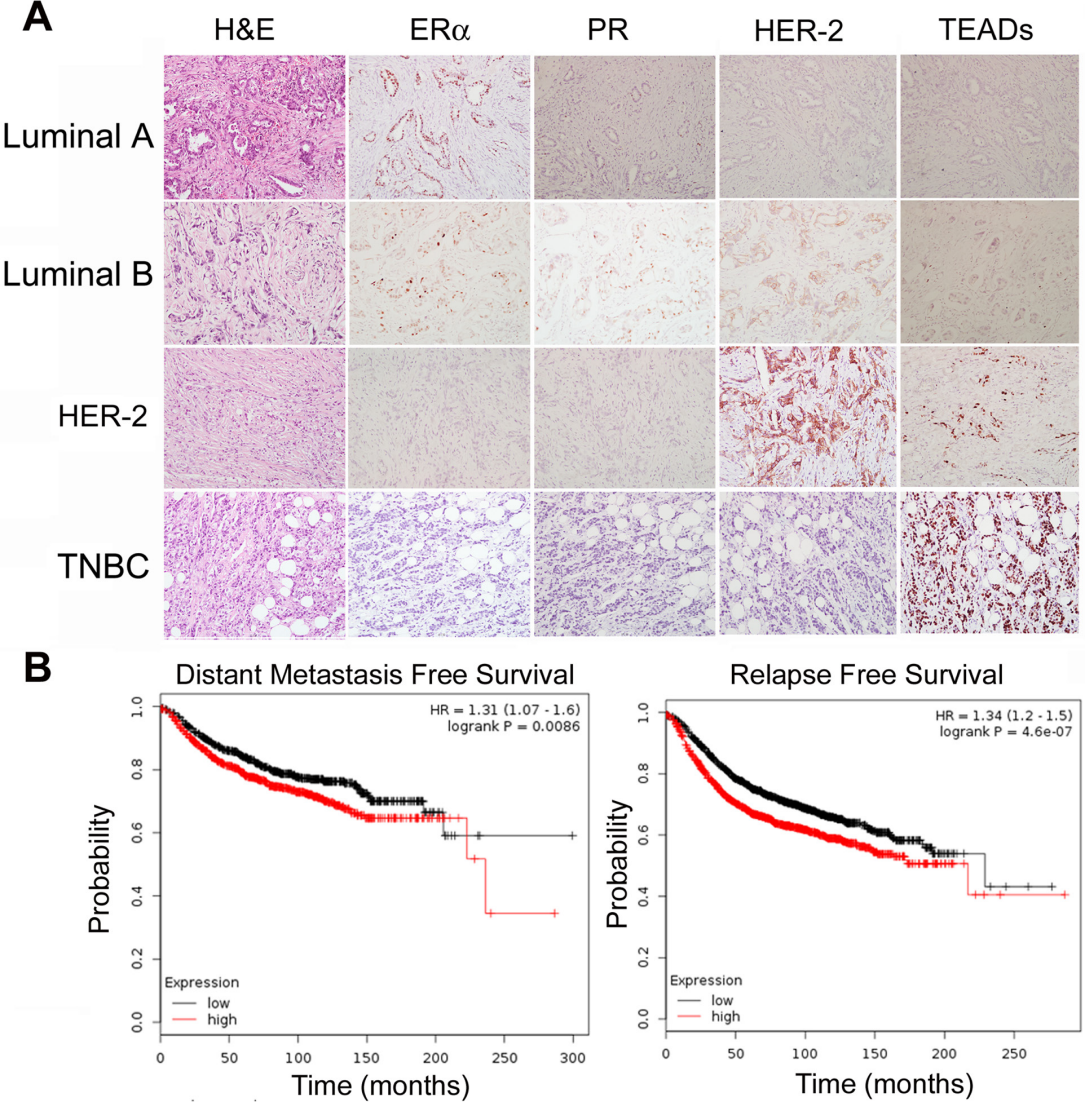
TEAD4 expression in human breast tumors **A.** 121 sample slides were stained with anti-ERα, PR, HER-2 and TEAD4 antibodies. Examples of IHC staining in breast carcinomas are shown. TEADs protein expression is negatively associated with the patient age. **B.** Kaplan-Meier plotter was used to analyze the breast cancer RNA seq data from the TCGA databse. High expression levels of *TEAD4* mRNA are significantly associated with patient distant metastasis free survival and relapse free survival.

IHC staining was used to test TEAD protein expression in breast tumors. We first validated TEAD1–4 antibodies for IHC using Flag-TEAD1–4 transfected HEK293T cells, but none of the anti-TEAD antibodies could distinguish TEAD1–4 via IHC (Figure S4). Despite this deficiency, we stained TEADs, ERα, PR, and HER-2 in 121 primary breast tumors (examples of IHC results are shown in Figure 6A). The expression of TEADs was detected in 15% of tumors examined (Supplementary Table 1), and TEADs expression negatively correlated with patient age (*p* = 0.009).

Since the anti-TEAD4 antibody did not specifically recognize the TEAD4 protein by IHC in clinical samples, we analyzed the *TEAD4* mRNA levels in breast tumors from the TCGA database. As shown in Figure 5B, high expression levels of *TEAD4* mRNA are significantly associated with distant metastasis and recurrence of breast cancer patients. These data suggest that the TEAD4 expression in breast tumors may be a poor prognosis biomarker.

## DISCUSSION

The TEAD transcription factor family contains four members (TEAD1–4) capable of forming transcription complex with YAP/TAZ to regulate the transcription of a number of downstream target genes [42]. While TEADs have been implicated in different cancers [43], the roles of TEADs in breast cancer have not been well studied. Among four members of TEAD, TEAD1 and TEAD4 are widely expressed in breast cancer cell lines, especially highly expressed in TNBC cell lines. Here, our present results from this study provide several lines of evidence supporting the putative role of TEAD4 as an oncogenic protein involved in breast cancer: (a) TEAD4 overexpression promoting DNA synthesis and tumorigenesis in HCC1937 cells; (b) TEAD4 depletion in HCC1806 and HCC1937 suppressing DNA synthesis, cell migration, and tumorigenesis; (c) TEAD4 suppresses the CDK inhibitor *p27* gene transcription together with KLF5, an oncogenic transcription factor; and (d) Depletion of *p27* significantly rescued the TEAD4 and KLF5 knockdown-induced growth arrest. Collectively, these findings provide novel evidence suggesting that TEAD4 promotes TNBC growth both *in vitro* and *in vivo*.

Alongside TEADs, results from the current study as well as from one of our previous studies demonstrated that KLF5 is highly expressed in basal TNBC cell lines and interacts with YAP/TAZ [12, 18], similar to TEAD1/4. Here, we examined whether KLF5 interacts with TEADs and found that only TEAD4 interacted with KLF5, working together to suppress the transcription of the *p27* gene. It is well established that normal cell contact inhibition activates the Hippo pathway and induces YAP phosphorylation [44] and *p27* [45]. Cytoplasmic translocation of pYAP may release the *p27* transcription inhibition mediated by TEAD4 and KLF5. Knockdown of TEAD4 and KLF5 together additively induced the expression of *p27* (Figure 4F) and TEAD4 could not efficiently suppress the *p27* expression without KLF5 (Figure 2E) suggest that TEAD4 and KLF5 collaborate to suppress the *p27* gene transcription in TNBC cells. However, it is worth noting that both TEAD4 and KLF5 may regulate the transcription of a number of target genes besides *p27*; for example, *p21* could be another target gene of TEAD4 and KLF5 (data not shown). Collectively, our results suggest that *p27* is an important target gene for TEAD4/KLF5 to promote cell proliferation because depletion of *p27* rescued the TEAD4/KLF5 knockdown-induced growth arrest.

Alongside our more conclusive findings regarding the role of TEAD4/KLF5, of our results are more suggestive and require further and more targeted follow-up. In particular, our present findings highlight the complexity of TEADs expression in breast tumors. Although four antibodies against TEAD1–4 worked well for WB (Figure 1A), they failed to specifically detect TEAD1–4 by IHC (Figure S4). For example, the anti-TEAD4 antibody also detected overexpression of Flag-TEAD1–3 in HEK293T cells, indicating that we detected all TEAD proteins in tumor specimens using this antibody in general. Similarly, we found that overall TEAD protein expression (using anti-TEAD4 antibody) was negatively associated with the patient age among the 121 primary breast tumor samples. The bioinformatic analysis from the TCGA database suggested that high levels of *TEAD4* mRNA are associated with metastasis and recurrence of breast cancer patients (Figure 6B). This is consistent with that TEAD4 promotes cell proliferation and migration. These results may be significant, but since they are rather general we suggest that more study is needed to develop more specific antibodies to better elucidate the protein expression of TEAD4 in breast tumor samples in clinic.

In conclusions, in the present study we demonstrated that TEAD4 is an oncogenic transcription factor promoting TNBC cell proliferation both *in vitro* and *in vivo*, with functional roles in the interaction between TEAD4 and KLF5 which suppresses the *p27* gene transcription. These findings suggest that TEAD4 may, in the future, potentially serve as a therapeutic target of TNBC.

## MATERIALS AND METHODS

### Cell culture and transfection

HCC1937 and HCC1806 TNBC cell lines (ATCC, Manassas, VA) were maintained in Dulbecco's Modified Eagle's Medium (DMEM) that contained 5% fetal bovine serum (FBS), 4.5 g/L glucose, 1 mM sodium pyruvate, 1.5 g/L sodium bicarbonate, 0.1 mM MEM nonessential amino acids, 4 mM L-glutamine, and 1% penicillin/streptomycin (P/S). The immortalized breast cell line MCF10A and 184A1, ERα positive breast cancer MCF7 and T47D, HER-2 positive cell line BT474 and SKBR3, and human embryonic kidney cell line 293T (HEK293T) were cultured as described previously [21, 22]. Lipofectamine 2000 (Invitrogen, Carlsbad, CA) was used to transfected siRNA and plasmids.

All siRNAs were purchased from Riobobio (Guangzhou, China). The target sequences for TEAD4 are 5′-GGACACUACTC TTACCGCA-3′, 5′-CCCATGATGTGAAGCCTTT-3′, 5′-AGACAGAGTATGCTCGCTAT-3′, and 5′-CTGTGCA TTGCCTATGTCTT-3′. The target sequence for *p27* is 5′-GGAGCAATGCGCAGGAAUATT-3′ [36]. The final concentration for pooled TEAD4 siRNAs and *p27* siRNA was 20 nM.

### Antibodies and plasmids

The anti-TEAD1 antibody (Cat# 610922) and anti-*p27*^Kip1^ antibody (Cat# 610241) were purchased from BD bioscience (San Diego, CA), the anti-TEAD2 antibody from GeneTex (Cat#GTX47542, Irvine, CA), while the anti-TEAD3 antibody (Cat# sc- 102130) and anti-TEAD4 (Cat# sc-101184) antibody were obtained from Santa Cruz Biotechnology (Santa Cruz, CA). The anti-KLF5 antibody used in this study was described in one of our previous studies [37]. The anti-Flag (Cat# F9291), anti-GST (Cat# G7781) and anti-β-actin (Cat# A5441) antibodies were obtained from Sigma-Aldrich (St. Louis, MO).

The human TEAD4 gene was amplified from MCF10A cDNA with the pfu enzymes via PCR using the primers 5′-GATCGGATCCTTGGAGGGCACGGCCGGCAC-3′ and 5′-GATCGAATTCTCATTCTTTCACCAGCCTGT-3′. The PCR products was digested by Bam HI/Eco RI and subcloned into the pBabe vector and verified by DNA sequencing.

### Immunoprecipitation (IP)

The full length *TEAD4* gene was cloned into the p3 × Flag-Myc-CMV-24 vector, and the HEK293T cells were transfected with TEAD4 and KLF5 expression constructs. The co-IP and GST pull-down experiments follow the protocols described in our previous studies [38, 39].

### DNA synthesis and cell viability assays

Cell proliferation rates of HCC1937 and HCC1806 cells were measured using Click-It EdU Alexa Fluor 488 and 647 Imaging Kits (Invitrogen) following the manufacturer's protocols. The cells were serum starved for 36 hours and stimulated with 10% serum for 4 hours. Cell viability was measured using Sulphorhodamine B (SRB) assays [40]. In brief, cells were fixed using 10% trichloroacetic acid (TCA) and stained with 0.4% SRB. After dissolving SRB from the available cells using 10 mM unbuffered Tris-base, optical results were read by an automated spectrophotometric plate reader set to a single wavelength of 530 nm.

### TEAD4 stable knockdown cell lines

A lentiviral pSIH-H1-Puro vector was used to express shRNAs, following the manufacturer's protocols. The TEAD4 shRNAs were designed according to the TEAD4 siRNA target sequences. Luciferase shRNA was used as a negative control. Lentiviruses were collected at 72 h post-transfection and used to transduce HCC1806 cells. At 48 h post-transduction, drug-resistant populations were selected using puromycin (2 μg/ml).

### Luciferase reporter assay

The 2-kb human *p27* gene promoter was cloned into pGL3-BASIC luciferase reporter vector using the following primers: 5′-TTCTCGAG AACCACAGGGTCCCGAGGGTC-3′ and 5′-TTAAG CTTCGTCCATCCGCTCCAGGCTAG-3′. HCC1806 cells were then seeded in 24-well plates at 1.5 × 10^5^ cells/well. At 24 h following plating, the cells were transfected with the *p27* promoter reporter plasmid, an internal control pRL-TK and the pooled TEAD4 siRNAs in triplicate. At 48 h post-transfection, the cells were collected and luciferase activities were measured using a dual luciferase reporter assay (Promega, Madison, WI) following the manufacturer's protocols.

### Quantitative RT-PCR

Total RNA was isolated using TRIzol (Invitrogen) and reverse-transcribed into cDNA with a RevertAid™ First Strand cDNA Synthesis Kit (Life Science, Shanghai) before being subjected to quantitative real-time PCR with gene-specific primers on an 7900 Fast Real-Time PCR System using SYBRGreen Master PCR mix (Life Technologies). GAPDH served as the internal control. Primers used for detecting *TEAD4* and *p27* expression at mRNA level were as follows: 5′-AACAGCGTGCTGGAGAACTT-3′ (TEAD4-forward), 5′-CTCACTGGCTGACACCTCAA-3′ (TEAD4-reverse), 5′-AGACGGGGTTAGCGGAGCAA-3′ (*p27*-forward), 5′-TCTTGGGCGTCTGCTCCACA-3′ (*p27*-reverse).

### Migration assays

Migration was evaluated by scratch assays using 6-well plates and transwell assays using 24-well chemotaxis chambers (Corning cell culture inserts, 8 μm pore size). The cells were washed twice with PBS, resuspended in 100 μl serum-free medium, and added into the upper chambers. The lower chambers were filled with 600 μl medium containing 10% FBS. For the migration assay, after incubation for 24 h, the cells on the upper side of the transwell membranes were removed using a cotton swab. The cells migrating to the lower side of the membrane were fixed in 4% paraformaldehyde for 20 min at room temperature, stained with crystal violet for 30 min, washed 3 times with PBS and dried off. The crystal violet was dissolved with 500 μl 33% acetic acid, and the OD570 value was recorded.

### Tumorigenesis in mice

For the HCC1937 xenograft tumor growth experiment, a total of 12 female NOD SCID mice of 5 weeks of age (Vital River, Beijing) were randomly divided into 2 groups that were injected with either HCC1937-pBabe or HCC1937-TEAD4 cells (3.5 × 10^6^ cells/point subcutaneously). For HCC1806 xenograft tumor growth experiment, a total of 12 female nude mice of 5 weeks of age were divided into 2 groups and injected with either HCC1806-Lucsh or -TEAD4sh cells (1.7 × 10^6^ cells/point subcutaneously). Tumor sizes in all four groups were measured twice per week for 3 weeks using Vernier calipers once tumors became palpable. Tumor volumes were calculated using the following equation: tumor volume (cm^3^) = (length × width^2^)/2. All mice were sacrificed at the end of the experiment and tumors were harvested and weighed.

### TEAD4 immunohistochemistry (IHC) staining

Anti-TEAD1–4 antibodies were validated for IHC using myc-TEAD1 or Flag-TEAD2–4 transfected HEK293FT cells (Figure S4). The anti-TEAD4 antibody (1: 100 dilution) was used for IHC after optimization. For TEAD4 staining, a total of 121 breast cancer samples were collected from the First Affiliated Hospital of the Kunming Medical University. A standard IHC protocol was performed as previously described [16]. Immunostained slides were scored independently by two pathologists using the ‘Allred score’ method. The study was approved by institutional ethics committees of the First Affiliated Hospital of the Kunming Medical University.

### The ChIP assay

The ChIP assay was performed using the HCC1806 cells. The diluted DNA-protein complex (25 μg protein) was incubated with different antibodies (goat anti-KLF5 Ab, goat IgG, mouse anti-TEAD4 Ab (Abcam Cat#AB58310), mouse IgG, and anti-H3K4Me Ab (Histone H3 (tri methyl K4) antibody, Abcam) overnight at 4°C in the presence of herring sperm DNA and protein A/G beads. PCR was performed by using primers for the *p27* gene promoter (5′-GCATTAACTTTGGCTCAAAC-3′ (forward) and 5′-CATGTCCTAACGTCCGATAC-3′ (backward)).

### Statistical analysis

All statistical analyses were carried out using the SPSS 13.0 (SPSS, inc., Chicago, IL). Data were analyzed by Student's *t*-test (two tailed). The Pearson Chi-Squared test was used to examine the correlation between TEAD4 expression and other clinicopathological parameters in primary tumors. The logrank analysis was used to analyze the relationships between the *TEAD4* mRNA expression levels and breast cancer patient distant metastasis free survival and relapse free survival. *P* < 0.05 was considered to be significant. Error bars represent SD.

## SUPPLEMENTARY FIGURES AND TABLE LEGENDS


