# Supplementary material for: The interplay between TEAD4 and KLF5 promotes breast cancer partially through inhibiting the transcription of p27Kip1
Source: Oncotarget. 2015 Apr 22;6(19):17685–97. doi: 10.18632/oncotarget.3779 (PMC4627338; doi:10.18632/oncotarget.3779)
Supplement: Supplementary file 1 [file oncotarget-06-17685-s001.pdf]

SUPPLEMENTARY FIGURES AND TABLE LEGENDS

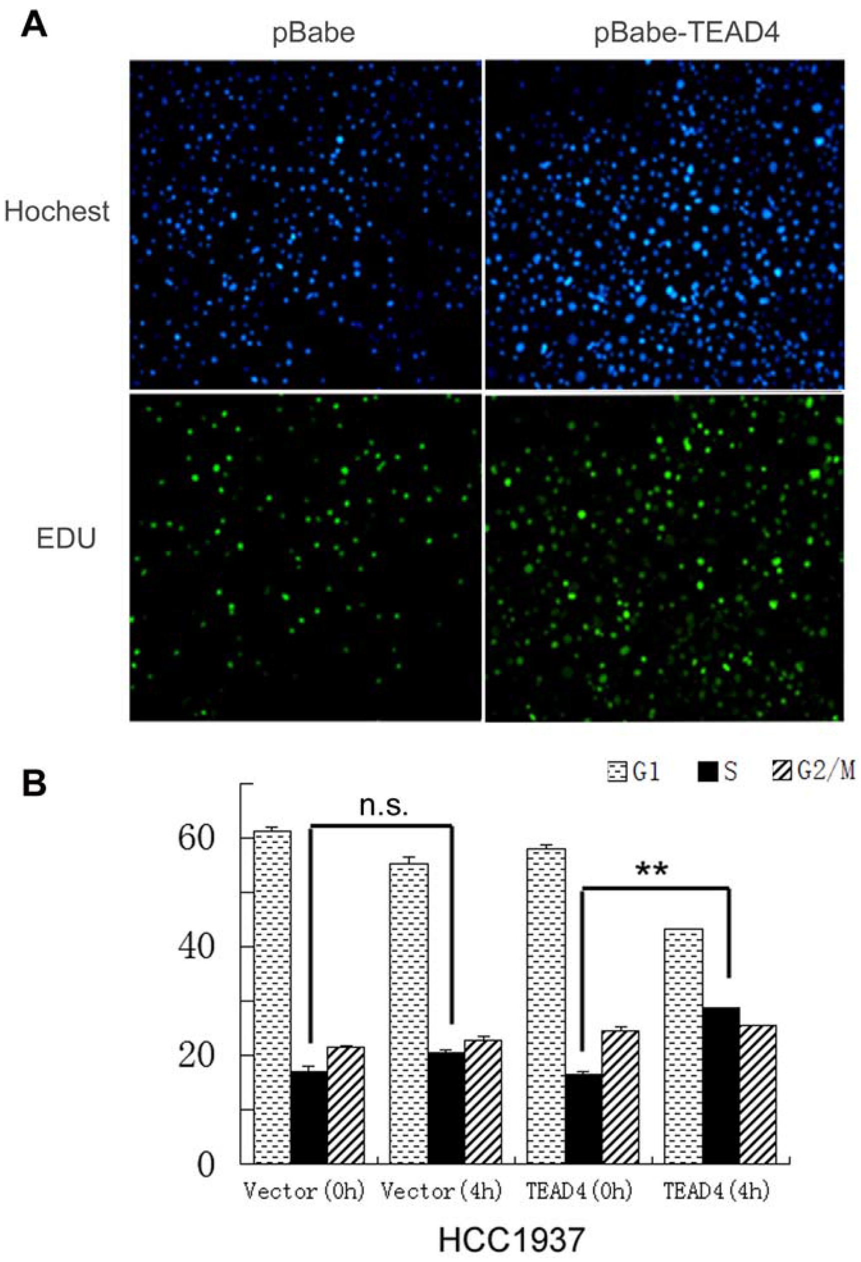

Supplementary Figure S1: TEAD4 overexpression promotes HCC1937 G1/S transition. **A.** TEAD4 overexpression significantly increased DNA synthesis in HCC1937 cells, as measured with a Click-iT EdU Alexa Fluor Imaging Kit. **B.** TEAD4 overexpression significantly increased G1/S cell cycle transition in HCC1937 cells, as measured by PI staining and flow cytometry. **, $p < 0.01$, n.s., not significant, *t*-test.

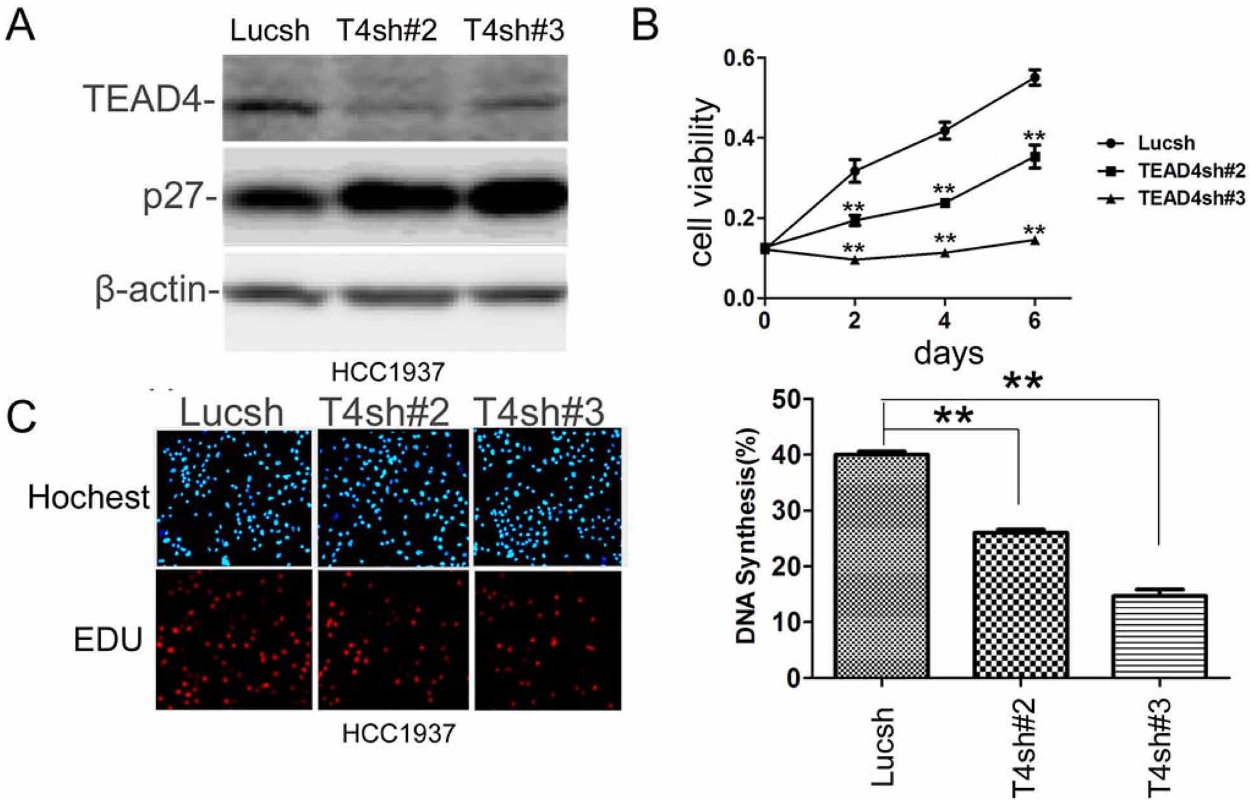

Supplementary Figure S2: TEAD4 knockdown inhibited HCC1937 cell proliferation. A. Stable knockdown of TEAD4 increased the *p27* protein level. **B.** TEAD4 knockdown significantly inhibited cell growth, as determined by the SRB assay. **$p < 0.01$, *t*-test. **C.** TEAD4 knockdown inhibited DNA synthesis by using the Click-iT EdU Alexa Fluor Imaging Kit. Quantitative results is shown on the right side, **$p < 0.01$, *t*-test.

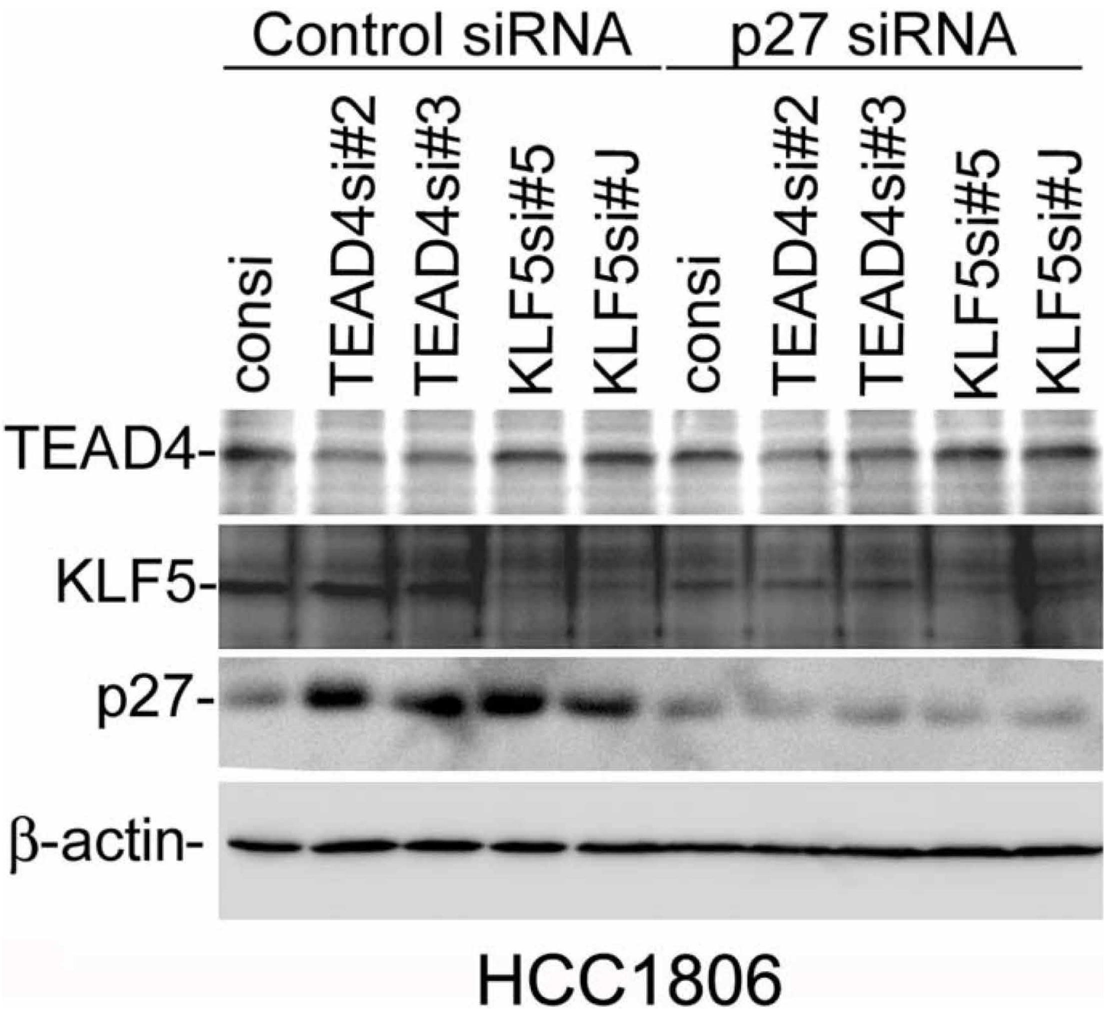

Supplementary Figure S3: TEAD4 and KLF5 promotes HCC1806 cell proliferation through suppressing the *p27* expression. Knockdown of TEAD4, KLF5 and *p27* in HCC1806 were evaluated by WB.

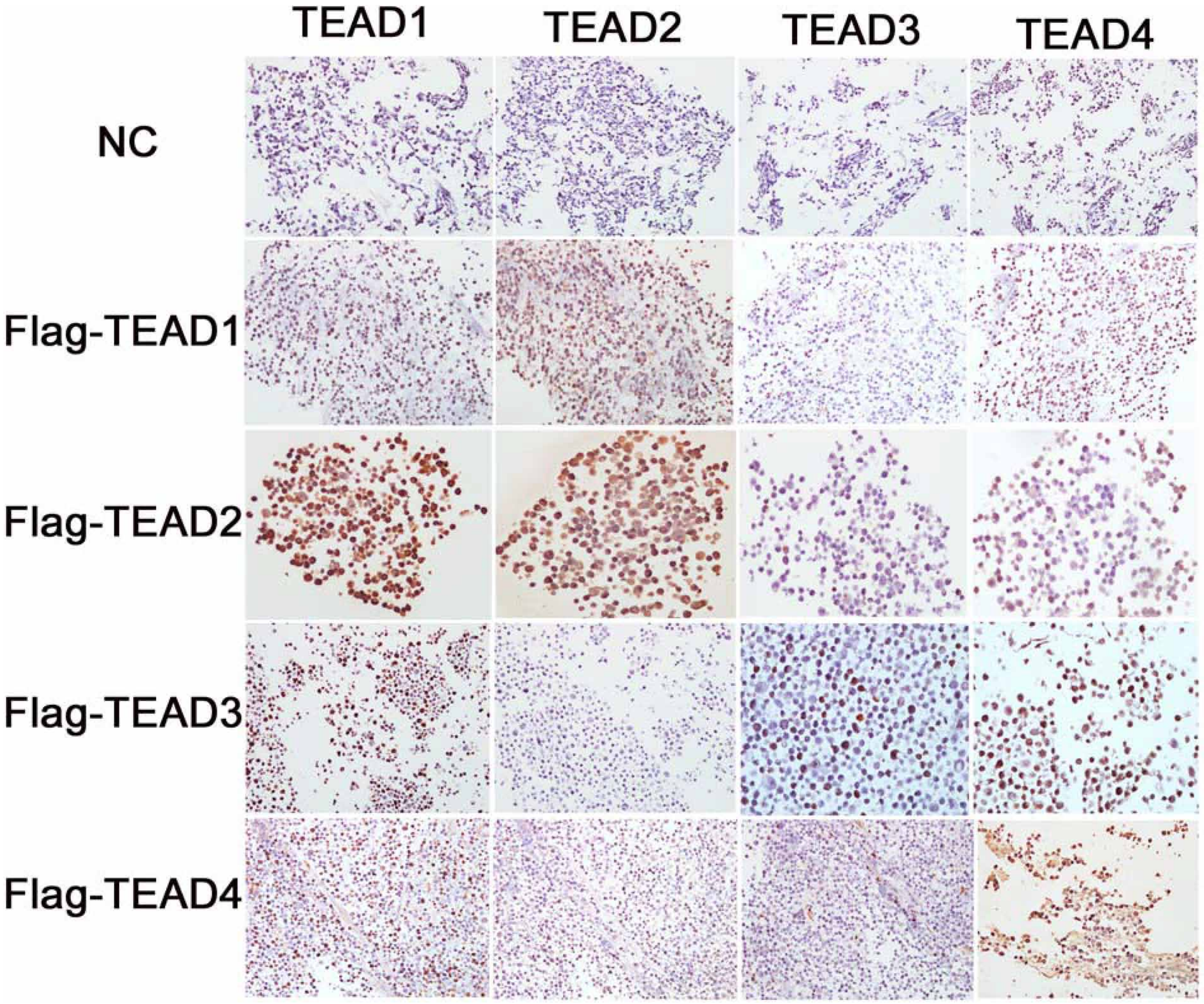

Supplementary Figure S4: Evaluation of anti-TEAD antibodies for IHC. Flag-TEAD1–4 was overexpressed in HEK293FT cells (Figure 1B) anti-TEAD1 and anti-TEAD4 antibodies detected all Flag-TEAD1–4 proteins by IHC.

Supplementary Table S1: Association between TEAD immunoreactivity and patient clinicopathological parameters

	Parameters	*N*	TEAD+	TEAD–	*p*-value
**Age**	<50 years	55	14	41	**0.009**
	≥50 years	43	2	41	
**Histological grade**	1	8	2	6	**0.876**
	2	34	4	30	
	3	40	9	31	
**ERα**	+	65	7	58	**0.221**
	-	55	11	44	
**PR**	+	62	9	53	**0.905**
	-	58	9	49	
**HER-2**	+	31	5	26	**0.246**
	-	89	13	76	

The anti-TEAD4 antibody was used to stain TEAD proteins in breast tumors.
